# Understanding the role of the volunteer in specialist palliative care: a systematic review and thematic synthesis of qualitative studies

**DOI:** 10.1186/1472-684X-13-3

**Published:** 2014-02-10

**Authors:** Rachel Burbeck, Bridget Candy, Joe Low, Rebecca Rees

**Affiliations:** 1 Marie Curie Palliative Care Research Unit, UCL Mental Health Sciences Unit, University College Medical School, 67-73 Riding House Street, London, UK; 2 EPPI-Centre, Social Science Research Unit, Institute of Education, University of London, London, UK

**Keywords:** Palliative care, Volunteers, Role, Systematic review, Thematic synthesis, Qualitative studies

## Abstract

**Background:**

Volunteers make a major contribution to palliative patient care, and qualitative studies have been undertaken to explore their involvement. With the aim of making connections between existing studies to derive enhanced meanings, we undertook a systematic review of these qualitative studies including synthesising the findings. We sought to uncover how the role of volunteers with direct contact with patients in specialist palliative care is understood by volunteers, patients, their families, and staff.

**Methods:**

We searched for relevant literature that explored the role of the volunteer including electronic citation databases and reference lists of included studies, and also undertook handsearches of selected journals to find studies which met inclusion criteria. We quality appraised included studies, and synthesised study findings using a novel synthesis method, thematic synthesis.

**Results:**

We found 12 relevant studies undertaken in both inpatient and home-care settings, with volunteers, volunteer coordinators, patients and families. Studies explored the role of general volunteers as opposed to those offering any professional skills. Three theme clusters were found: the distinctness of the volunteer role, the characteristics of the role, and the volunteer experience of the role. The first answers the question, is there a separate volunteer role? We found that to some extent the role was distinctive. The volunteer may act as a mediator between the patient and the staff. However, we also found some contradictions. Volunteers may take on temporary surrogate family-type relationship roles. They may also take on some of the characteristics of a paid professional. The second cluster helps to describe the essence of the role. Here, we found that the dominant feature was that the role is social in nature. The third helps to explain aspects of the role from the point of view of volunteers themselves. It highlighted that the role is seen by volunteers as flexible, informal and sometimes peripheral. These characteristics some volunteers find stressful.

**Conclusions:**

This paper demonstrates how qualitative research can be sythnesised systematically, extending methodological techniques to help answer difficult research questions. It provides information that may help managers and service planners to support volunteers appropriately.

## Background

Volunteers are integral to the hospice movement in North America [1-3], India [2], Uganda [3], and in several European countries, including the UK [4-7]. Demand for end-of-life care is increasing worldwide with more people dying annually (predicted rise from 2002 is between 14% and 42% by 2030 [8]). Many of these deaths follow a period of chronic illness, such as cancer, heart disease, stroke, and chronic respiratory disease, which may involve palliative care in the advanced stages [9]. In the US there are around 400,000 hospice volunteers [10]. In the UK, where hospice care can include inpatient and day care services (services provided in a hospice building to non-resident patients), and home-based care, there are more than 100,000 volunteers [11], and their contribution reduces hospice costs by an estimated 23% [12]. Volunteers are therefore vital to hospice services.

Whilst we are only beginning to gain a picture of volunteers’ activities in relation to tasks undertaken in specialist palliative care, particularly those in patient-facing roles [13,14], it is clear that volunteer contributions go beyond the purely physical. For instance 86% of hospices involving volunteers in roles with inpatients reported that the volunteers provided emotional care [13]. Although this kind of contribution can be hard to quantify, it appears tangible to those involved [15]. Similarly, whilst a job description lists the activities that someone fulfilling a position is expected to undertake, the actual role is likely to involve other elements which might be harder to describe. This role in the minds of those involved (both those undertaking the role, and others in contact with people undertaking the role) might also be distinct in other ways from other roles within an organisation. Roles can be understood based on three concepts: as a patterned and characteristic social behaviour, as an identity assumed by a social participant, and as a script or expectation for behaviour that is understood by all and adhered to by the performer involved [16].

One aim of qualitative research is to discover what a phenomenon is like from the point of view of those experiencing it, thus providing access to an insider perspective [17]. A narrative review of such studies would bring the research findings together by describing and comparing them. However, more sophisticated techniques are being developed to synthesise this literature. These apply the rigour of systematic reviewing, but at the same time, are sympathetic to qualitative research methods and ‘go beyond’ a mere description of findings [18]. These methods aim to be interpretative and configurative (i.e., aiming to make new or enhanced meanings by making connections between existing studies) rather than merely adding together or aggregating the qualitative study findings [19,20]. Reviews using these techniques include topics such as lay understanding of cancer risk [21], and children’s and young people’s perspectives on body size [22,23].

We undertook such a review of perspectives on the volunteer role in specialist palliative care, focusing on the role of volunteers with direct patient and family contact, and using thematic synthesis [24]. We aimed to uncover how the volunteer role is understood by those experiencing the role (either as volunteers, volunteer coordinators or patients and family carers), rather than just what volunteers do.

## Methods

### Identifying papers for review

#### Inclusion criteria

We included qualitative studies which provided data on the experience of being, or receiving care from, a volunteer in end-of-life care, based on pre-defined inclusion criteria (see Table 1).

**Table 1:** Study inclusion criteria

***Inclusion criteria***	
**1.**	**Study design** qualitative study, defined as:
	○Data collected by talking to participants, for example, using in-depth or semi-structured interviews and/or focus groups
	○Employing a qualitative data analysis method, either mentioned by name, such as grounded theory, discourse analysis, or interpretative phenomenological analysis, or indicated by the use of words associated with such techniques, such as hermeneutic, thematic, or inductive.
	○The presence of at least one of the above criteria (data collected or analysis method) should be indicated in the abstract
**2.**	**Study population** participants (i.e., people providing the data) should have direct experience of volunteering or of others volunteering in end-of-life care, typically volunteers themselves, patients, patients’ (including deceased patients’) families/carers, or end-of-life staff. This should be clear from the abstract.
**3.**	**Data #1** there must be extractable data about the role of volunteers in end-of-life care from participants’ point of view, for example, but not limited to, what they understand the role of end-of-life volunteers to be, what a volunteer in end-of-life means to them, or what is the contribution of volunteers in end-of-life care. Check full paper.
**4.**	**Data #2** Data relating to volunteers can be extracted separately from that relating to other groups, such as paid staff. Check full paper.
**5.**	**Care focus #1** examines end of life care, defined as care for people at the end-of-life, regardless of age or disease, including palliative care, in any setting (eg hospice, palliative care unit, home care). This should be clear from the abstract, but if in doubt, check full paper.
**6.**	**Care focus # 2** focuses on care given by volunteers in patient/family-facing roles. Should be clear from the abstract, but if in doubt, check full paper. Care defined as activities volunteers perform in relation to patients/families or their implicit role in relation to patients/families.
**7.**	**Reporting language** is reported in English. Clear from full reference.
**8.**	**Report format** is not a dissertation or thesis or conference abstract. Clear from full reference.

### Search process

We searched from inception to May 2013 the following electronic databases: Amed, Cinahl, Embase, Medline, PsycInfo, Science Citation Index Expanded, Social Sciences Citation Index, Conference Proceedings Citation Index – Science, Conference Proceedings Citation Index - Social Sciences & Humanities, IBSS, Campbell Library, and OpenGrey (Sigle). As part of the searches for a wider project on volunteer activity in palliative care ( http://www.mariecurie.org.uk/Documents/Research/funding-research/bridget-candy-short-grant-summary.pdf), we searched for all available literature (regardless of research method) reporting empirical research on volunteers in palliative care settings. We developed search strings for ‘volunteer’ and ‘palliative’ using both free-text terms and subject headings tailored to each database searched (Additional file 1). In addition, we searched abstracts from relevant conferences and searched all abstracts available online from the Journal of Pain and Symptom Management and Palliative Medicine. We scanned reference lists of studies meeting our inclusion criteria, and undertook forward citation searches of included studies. We identified researchers in the field using a snowball technique, and contacted those found for information on unpublished or soon-to-be-published studies.

### Study selection

Two reviewers independently scanned titles and abstracts of downloaded citations to identify papers potentially relevant to our broad topic area of volunteers in palliative care settings (RB, BC). The outputs of the two reviewers were combined, and inconsistencies discussed to resolve differences. From the resulting list, studies were identified for potential inclusion in the present review based on the above inclusion criteria. We retrieved the full papers for citations which appeared to meet our inclusion criteria, and double-checked that the criteria were in fact met.

### Quality assessment

For papers that met our inclusion criteria, we undertook two quality assessment procedures since we could find no single tool which both covered practical issues involved in qualitative research (mediated by the quality of reporting rather than necessarily a true reflection of what researchers actually did) and looked at the findings and overall usefulness of potential studies in sufficient detail. First, we applied the Critical Appraisal Skills Programme (CASP) [25] qualitative appraisal tool, which focuses on the rigour, credibility and relevance of research. Second, we used the method developed for another qualitative systematic review [22] which was modified from a set developed for examining the findings of evaluations of intervention processes [26,27]. This method focuses in more depth on both the reliability and usefulness of a study’s findings (available from the authors).

### Analysis methods

We used the method for thematic synthesis outlined by Thomas and Harden [24]. This involves three stages: coding the text, developing descriptive themes, and generating analytical themes.

We used EPPI-Reviewer 4 software to organise codes into hierarchical structures, and to keep track of study details and the quality assessment process [28]. The text of each included study was loaded into the software verbatim. One reviewer (RB) developed a set of descriptive codes inductively by coding each line of the findings sections of all the included studies, focusing on those portions of study findings that met our inclusion criteria, categorising meaning and content. Working independently, a second reviewer (BC) using Word software re-applied the codes developed by the first reviewer to the study texts, and, in the process, developed additional codes. The two reviewers discussed the codings, and the first reviewer re-examined the entire set of findings again, paying attention in particular to the new codes identified by the 2^nd^ reviewer, updating the coding frame where they agreed with interpretations.

Initial codes were grouped and regrouped so as to create themes, and then hierarchies. The groupings were then further refined by discussion (RB/BC) and re-checking of the original studies (RB/RR). Successive drafts of a narrative that described the themes seen in the findings were then discussed by the wider study group (RB, BC, JL, RR) and further refined.

## Results

### Studies found

**Figure 1:**
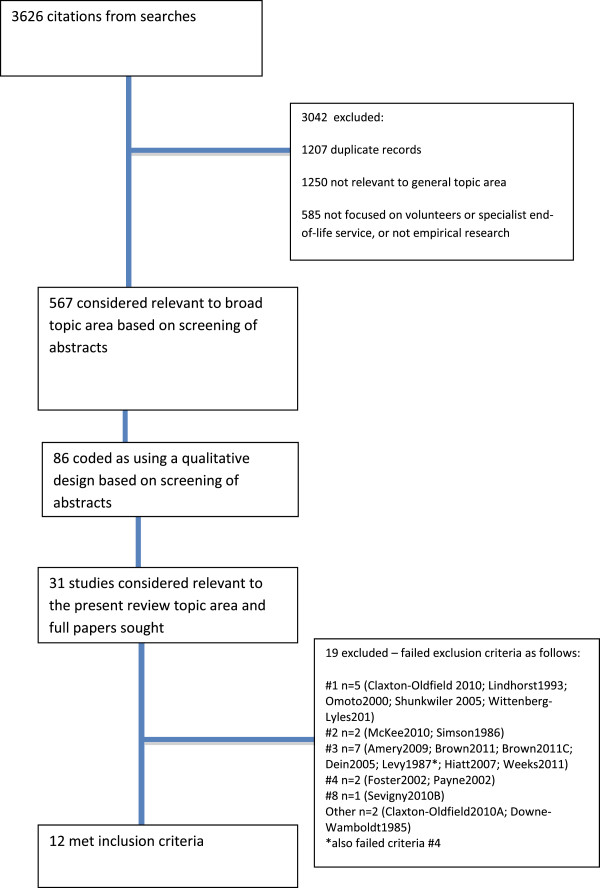
Flow chart of studies through the review process.

In all, 3626 citations were found in searches, with 31 full-text reports retrieved because they appeared potentially relevant to the present review, of which 19 were excluded at full text. The flow of studies through the search process with reasons for exclusion can be seen in the flowchart (See Figure 1).

### Included studies

Twelve studies met our inclusion criteria [1,3,5,29-37] involving 294 participants. In three studies respondents had experience of care based in a dedicated hospice building [29,31,36], in six respondents had experience of home-care [31,33-35,37], and in three respondents had experienced a mix of care settings [1,30,32]. In seven studies respondents were volunteers [1,29-33,36] (168 participants with ages ranging from 18 to 74), in two they were patients and/or family members of patients who had received volunteer care [5,37] (38 participants, age range 23-87), and in three respondents came from a mixed population from different care settings or were volunteer coordinators [3,34,35] (130 participants, age range 28-65+). The majority of participants in all studies were women. All but three studies [1,32,33] were published since 2004. Seven studies were from North America [1,29,30,32-34,37], four from Europe [5,31,35,36] and one Africa [3]. See Additional file 2 for further details. Comparisons between studies based on setting are drawn where appropriate in the discussion. Other comparisons, for example, between different groups of respondents, have not been made since there were relatively few studies in some categories.

Analysis methods used included thematic analysis [3,30-32,35,36], hermeneutic approaches [29,37], and grounded theory [1,34]. Two studies did not state the analysis method used [5,33].

### Quality assessment

A summary of the quality assessments can be seen in Table 2. On the CASP criteria eight studies met at least eight of the nine criteria, but three studies met fewer than this [5,32,33]. The criteria most commonly not met were adequate consideration of the relationship between researcher and participants, consideration of ethical issues, and sufficiently rigorous data analysis. The included studies followed a similar pattern when appraised with the second set of quality criteria, with the same studies scoring lower than the others [5,32,33]. Only six studies met at least three of the four criteria on both scales (reliability/trustworthiness and usefulness). However, two studies, which met all of the CASP criteria, did not score well on reliability and trustworthiness of findings on the second set of criteria [35,36]. We did not remove studies from the synthesis if they scored poorly (not defined *a priori* since no agreed cut-off has been set) on the quality assessment criteria, preferring instead to compare their contribution to the analysis with that of other studies. Further details can be seen in Additional files 3 and 4.

**Table 2:** Summary of the quality assessment process

**Study**	**Number of CASP criteria met (maximum 9)**[25]	**Additional assessment criteria**[26,27]**: Overall reliability/trustworthiness of the findings**	**Additional assessment criteria**[26,27]**: Overall usefulness of the study**
Andersson [29]	8	3/4	3/4
Berry [30]	8	3/4	2/4
Field-Richards [31]	8	3/4	3/4
Finn-Paradis [32]	6	2/4	3/4
Guirguis-Younger [1]	8	3/4	3/4
Harris [33]	3	2/4	2/4
Jack [3]	8	3.5/4	3/4
Luijkx [5]	6	2/4	2/4
McKee [34]	9	3/4	3/4
Sevigny [35]	9	2.5/4	3/4
Watts [36]	8	2.5/4	4/4
Weeks [37]	8	4/4	4/4

### Synthesis findings

The findings are grouped around three theme clusters: the distinctness of the volunteer role, the characteristics of the role, and the volunteer experience of the role (see Table 3). Although they overlap to some extent the first answers the question, is there a separate volunteer role? The second helps to describe the essence of the role, and the third helps to explain aspects of the role from the point of view of volunteers themselves. Additional file 5 shows themes found by study and Additional file 6 shows a sample sub-theme with its constituent codes. No single study contained all the sub-themes, although all studies contributed to all three theme clusters.

**Table 3:** Theme clusters found in the synthesis

**Theme cluster**	**Sub themes**
Distinctness of the volunteer role	Distinct role from that of staff
	Specific volunteer roles: professional-like; go-between; advocate/mediator; teamwork; surrogate; relationship roles
Characteristics of the role	Social nature of the role
	Providing support
	Just being there
	Just listening
	Keeping patients happy
Volunteer experience of the role	Ambiguity, flexibility and informality
	Staff restrict information
	Staff control the volunteer role

### The distinctness of the volunteer role

Study participants, who included patients and families, as well as volunteers themselves, saw the volunteer role as having an identity separate from the role of paid staff. They also distinguished specific volunteer roles.

### Distinct role from that of staff

Respondents made distinctions between staff and volunteer roles by talking in terms of recognised work boundaries [29,34,35]. The language used included "*not doing someone else's ‘paid work’, not stepping on the toes of professionals."*[34], p.167 and "*being afraid to cross over into the domain of the nurses"*[29], p.605.

Emphasis was also placed on the ways specific tasks and kinds of relationship were different for volunteers compared with staff. One study of hospice volunteers reported that they might be asked to stay for longer hours, "*as one might ask of a friend but not of a professional…."*[30], p.461. In another, a bereaved spouse said,

*"And I think, for Matthew, the fact of having somebody from outside, not just staff, is important. I think the staff that deal with you all the time, there is some humiliation in your situation that staff has to deal with at another level, his physical needs, so this is strictly someone to talk and be there, a friendly face, a kind face"*[37], p.89.

Participants also emphasised the distinctiveness of volunteer roles from those of staff. In one study the volunteer role was summed up as ‘*complementary, not substitutive’*[35], p.743. Another study reported that volunteer coordinators considered that volunteers "*provide what no one else on the health care team provides—they are friends and companions to the dying - the possibility of infringing on the roles of others on palliative care team is minimal"*[34], p.167.

### Specific volunteer roles

Study participants described two categories of volunteer role: one characterised by independence, where volunteers undertook a task that might otherwise not be done by anyone else, and the other characterised in terms of surrogacy.

The first of these included acting as a ‘go-between’ between patients/family and paid staff [31,35], or acting as an advocate for the patient and family [33,35]. One volunteer reported how volunteers *"…might notice something that hasn’t been picked up by the nurses or something, indeed they might not have said to the nurses, something that they perhaps feel unable to talk about. So you know, we are able to pass stuff on if necessary"*[31], p.629.

The role of volunteers in representing patients’ interests is explicitly mentioned by volunteer co-ordinators in one study, who said, *"They [volunteers] also act as mediators and, at times, advocate for the patient"*[35], p.741.

When volunteer roles were talked of in terms of surrogacy, volunteers were seen as becoming, albeit temporarily, an additional or substitute family member or friend [31,33,34,37]. One study reported that participants talked of how "*… volunteers will step in …to forge a bond of real friendship so that when the client dies they are ‘like family’"*[34], p.166.

As one bereaved spouse put it, "*Knowing somebody else was aware of what the situation was. I think that was the biggest thing, other than the nurses in the hospital. It was just good. It was a replacement for family, because my family was all away"*[37], p.89.

### The characteristics of the role

#### The social nature of the role

The volunteer role was largely characterised in social terms rather than in terms of the specific tasks they undertake [5,29-32,34-37]. As one volunteer said*, "We encourage the other side of what obviously makes you human not just you know having your physical stuff seen to but just having the social stuff seen to*" [31], p.629.

Participants emphasised the importance of relationships, both actual and anticipated. One study reported that *volunteers* "*clearly voiced, that developing genuine and true relationships … constitutes one of their main functions*" [35], p.740. For others, experiences of the social side of their work had not always met expectations [29,32]. A volunteer in one study, for example was reported as saying they had expected *"more opportunities to meet them* [patients] *in some kind of human relationship"*[29], p.605.

### Providing support

Participants talked about the value of the social support that volunteers can provide, notably emotional support [5,32,37], and keeping patients happy [29,34,37], for example:

*"They* [the volunteers] *sat and talked to me* [bereaved spouse] *and just spent time with me, letting me do whatever I wanted to do until I could come to grips with the situation, so it was emotional and mental."*[37], p.90.

*"Most of the conversations were about everyday things: ‘to make things a little positive, it won’t be that sad then"*[29], p.606.

Accounts also emphasised the value of what might seem very basic social acts, using the word ‘just’ in phrases such as ‘just being there’ [1,32-35,37], and ‘just listening’ [30,31,34,37].

"*All of the coordinators explained that what their volunteers actually "do" is hugely variable and depends completely on what the client and family need and ask for.* ***What does not vary is a fundamental commitment to being there*** *"* [emphasis added by review authors] [34], p.166.

### Volunteer experience of the role

When volunteers described their experience of their role, their accounts referred to ambiguity, flexibility and informality. Central to their experiences were volunteers’ relationships with paid staff, and the control had by paid staff over volunteers’ work.

#### Ambiguity, flexibility and informality

***Ambiguity*** was connected either with uncertainty around the tasks volunteers should undertake [32], or to a lack of clarity about their role on the part of a volunteer in relation to other roles undertaken in their day-to-day life, for example, between being a volunteer and also knowing the patient as a neighbour [34]. This could have negative connotations, for example, *"35% (6) of the volunteers interviewed noted that they were not always clear about their role in the organization and, as a result, felt job stress"*[32], p.170.

***Flexibility*** of the role was seen not only in terms of fitting the tasks undertaken to each volunteer’s skills and interests [1,31,36], but also in terms of ‘doing what is needed’ [1,3,30,31,34,37]. Volunteers said, for example, *"[We do] the kinds of things no one else has time to do"*[34], p.166,

*"As long as what I am doing is help to them* [staff and clients]*, I am happy with what I am doing. It does not matter what the job is. ….. and as long as I am of assistance, I am happy . . . ."*[1], p.18.

Volunteers referred to the unpaid nature of their role and reported that this led to their perception of the role as informal [31,35,36]. A perception of ***informality*** was reported in one study to have "*attracted them* [volunteers] *to volunteer initially*" [31], p.628.

Informality was also mentioned as something that for some volunteers was slowly being eroded from the volunteer role as an increasingly structured working environment was being introduced, often as the result of a more rigorous legal framework [31,36].

*"It all has got so formal somehow and maybe we matter less because of that"*[36], p.112.

### Relationship of the volunteer role to that of staff

Some volunteers described aspects of their role that might be considered ‘professional-like’ [1,29,30,32]. This included using medical terminology [32] and approaching patients in a more formal way than a friend would [29].

Volunteers also emphasised the value of being part of a team with staff [1,29,31,32]. As one put it, *"I very much appreciate that we work as a team . . . it takes some time to develop a relationship with the nursing staff, but once you have that, you are definitely a part of a team"*[1], p.20*.*

### How staff control the volunteer role

In some studies participants indicated that the volunteer role is controlled by staff in a way which is not always appreciated by volunteers. This included reports of staff not planning in advance the tasks they would like volunteers to undertake, which volunteers found stressful [29]. One study described volunteer frustration with reporting arrangements that acted to limit their role: "*Several volunteers wanted to function as counselors* [sic] *and to communicate directly with the nurse rather than to work with the social worker. They perceived the nurse as having greater authority and power over patient care plans than the social worker"*[32], p.171.

In two studies volunteers acknowledged their role as less important than that of staff [29,32], although they wanted to know what was going on, for example:

*"I expect to be on the periphery, but I want to know what ingredients there are in what we are cooking here"*[29], p.605.

Volunteers implied that access to information would alleviate this:

*"As a volunteer I can easily feel left out since I do not understand much of what’s going on in the work"*[29], p.605.

The potential restriction by staff of volunteer access to patient information was a recurring theme [29,31,32]. Participants described concerns about appropriate activities for patients (for example, whether it was appropriate to take patients for a walk [29], p.606), patient confidentiality [32] and safety [31], as well as emphasising the need for information-sharing to improve team-work [29].

The control of volunteers by paid staff was sometimes described negatively [29,31,32]. In these studies, volunteers perceived that staff might feel insecure and/or threatened by them.

*"I don’t know why they would because I mean we’re not professional and you know we’re not nurses … I mean we’re only here to help you know…we’re not looking to take their jobs off them"*[31], p.629.

However, this was not always the case:

*"If there were things that I thought I wanted to do for the clients, staff would say go ahead, as long as it is safe. … It is a very open and supportive environment. If I said I wanted to try something, the staff would go to great lengths to help me get it started"*[1], p.18*.*

## Discussion

In this systematic review of qualitative studies we aimed to uncover how the volunteer role in end-of-life care is understood by those who are closely involved with volunteering, for example, by being a volunteer or receiving volunteer care. Our findings suggest that a volunteer role exists which is largely distinct from that of paid staff, and is broadly social in nature. The findings will now be discussed including some areas of contradiction.

### Distinct role versus the professional-like aspects of the role

Although the volunteer role seemed to be distinct from that of paid staff, the synthesis also found that volunteers in some studies appeared to take on a quasi-professional role. It may be that volunteers need to feel that their role is important in order to justify the time and effort they put into it and, therefore, they are keen for it to be seen as a distinct role. However, they may also feel that patients and families (and maybe the researchers) will take them seriously only if they appear to be professional-like. The concept of professionalism involves lay people putting their trust in professionals who in return maintain confidentiality [38]. Therefore, ‘professionalising’ the volunteer role in this way may be an important part of how patients and volunteers ‘negotiate’ their interaction. Volunteers’ taking on a professional-like role was more common in studies where volunteers were respondents, contrasting with studies where family members were respondents where volunteers were more likely to be cast in a surrogate family role. There was a similar contradiction between the description of the role as ‘complementary not substitute’ and the finding that volunteers ‘do what is needed’ (seen in the characteristic ‘flexibility’). This could imply that volunteers perform tasks which staff do not have time for, which suggests that the volunteer role may not really be a distinct role, but a way of stretching available funding. It is not clear from the included studies where participants talked about ‘doing what is needed’ what the tasks involved were, and whether they were part of paid staff’s roles or not. Further primary research, such as an ethnographic study following volunteers in relevant settings, could usefully explore all these anomalies.

### Types of role

The two types of volunteer role which emerged from the synthesis, independent roles and surrogate family roles, show how the volunteer role may adapt to the care setting in which it is played out. The included studies were undertaken in different settings, including patients’ homes and inpatient care facilities. Surrogate family roles arose most frequently in studies of volunteers in homecare or mixed (i.e. both homecare and inpatient) settings. This may be because in inpatient facilities volunteers appear to patients and families to be more like staff in a setting where paid staff were also working. In the home, where patients and families are in a familiar environment, volunteers may feel most comfortable taking on the role of family member or friend. This may be particularly the case where there has been contact over a longer period of time or where only one volunteer has been involved with a family as can be the case when volunteers provide care in patients’ homes. On the other hand, patients and families using inpatient facilities are likely to encounter relatively large numbers of staff and volunteers. Again, this contrast between roles could be usefully explored by further primary research, such as a qualitative study comparing relatives’ experiences of volunteers in different settings.

### Social nature

The dominant characteristic of the volunteer role emerging from the synthesis was its social nature, in particular that respondents characterised the role in social terms rather than in terms of the tasks they undertake. The perceived social aspect of volunteering in a hospice setting has been found to be a motivating factor for volunteers [39,40]. The social cluster of themes was found in studies interviewing volunteers and in those interviewing family members. It was also found in studies in both home- and hospice-based care, indicating that this is a fundamental aspect of how the volunteer role is understood. Forming relationships with patients and their families are an important and sometimes neglected aspect in palliative care [41], for example, palliative care patients receiving volunteer visits have been shown to live for longer than those not receiving visits [42].

### Volunteer experience

The synthesis also identified ambiguity in how the volunteer role is understood. On one hand the role was attractive to volunteers because of its flexibility and informality, qualities which were viewed positively by volunteers (although they are not defined in the studies, so it is unclear to what volunteers were specifically referring). On the other hand, volunteers found it stressful when the tasks to be undertaken were not clearly established, and when this was the case, the role was described as ambiguous. Since a largely quantitative study reports low levels of role ambiguity and role conflict among 97 hospice volunteers in Australia [43] this aspect of our findings would also be worthy of further primary research, such as a qualitative study focusing on volunteers’ perceptions of this aspect of the role. Given the increased formalisation of volunteer roles resulting, for example, from legislation around health and safety [44], these aspects may effect volunteers’ motivation and satisfaction with the role [45].

The synthesis highlighted the relationship between volunteers and paid staff as important in shaping the volunteer role. This included the way staff ‘control’ the role, for example, in some studies through restricting access to patient information. It is clearly expected that staff direct the volunteer role, for example, designating the tasks volunteers undertake, since they have overall responsibility for patient care. However, a quotation from a volunteer *"in one study I expect to be on the periphery"*[29] suggests that this volunteer sees their role as subordinate to that of staff (although the quotation begs the question, periphery of what?). The synthesis also identified issues of staff insecurity, as well as examples where members of staff were supportive of volunteers. All of these findings suggest that power relationships may shape the volunteer role [46], p.154, and further primary research exploring this would be worthwhile, for example, a qualitative study focused on the relationship between volunteers and staff.

In a previous survey, we found that some people volunteered their professional skills, most commonly hairdressers and beauty therapists [13]. In the studies included in the present review, authors did not differentiate between volunteers offering professional skills and others, implying that none of the volunteers were offering professional skills, although this is not explicitly stated. A study specifically of volunteers offering professional skills and comparing their experiences with those of other volunteers might be of value to volunteer coordinators to help manage both groups more effectively.

### Implications of the findings

Understanding how a role is conceived by those involved (in the present review mostly volunteers, but also some family members and volunteer managers/coordinators) will help planners and managers of palliative care services to make the most of volunteers’ contribution. For example, our findings could help volunteer coordinators present the role as realistically as possible to prospective volunteers. In particular, understanding that the role is not simply a set of tasks to be undertaken and that the role is strongly social in nature is important. Also, an appreciation of how the role is understood will be helpful in training paid staff to work alongside volunteers as effectively as possible. A specific issue which could be addressed includes the contrast between ambiguity in the role, which was stressful for volunteers, and flexibility, which volunteers liked (although this may be being eroded by increasing formalisation of the role due to the introduction of management policies, such as job descriptions [44]). Also, it would be useful to help volunteers understand and deal with any ambiguity they may experience in their role, as well as reducing any ambiguity that may exist by training staff to understand what volunteers can contribute to the patient and family experience of care.

### Methodological reflections

#### The quality assessment process

Three studies contributed very little to the analysis [3,5,33]. One of these scored poorly on both quality assessments [33], and one other [5], scored poorly on one set of criteria [22] and lower than most of the other studies on the other [25]. Retaining them in the analysis did not appear to compromise the synthesis since the weaker studies merely contributed to fewer themes rather than changing the themes. The third study [3], although scoring well on both quality assessments, differed in other ways from the other studies. It was the only study not undertaken in a developed country, and volunteers in this study undertook different tasks compared with those in the other studies (for example, providing clinical care). Its focus was also more on what volunteers do rather than on what it was like either to be a volunteer or to receive volunteer care. Furthermore, although this study’s authors collected qualitative data, their analyses which used content analysis, were less inductive than most of the techniques used in the other studies. It should also be noted that the CASP criteria rely on quality of reporting which, in turn, is affected by publisher word limits, so not meeting these criteria may have been because of inadequate reporting rather than because these issues were not considered by study authors.

Turning to the second quality assessment tool used [22,26,27], which focuses in addition on the usefulness of a study’s findings, three studies scored two points out of a maximum of four for ‘overall usefulness’ [5,32,33] which included an assessment of the extent to which the study privileged the role of volunteers. In one of these the focus was on volunteer stress and burnout [32]. In another, which is a comparison of paid and unpaid workers, the findings included little material suitable for synthesis [33]. In the third study the material presented focuses more on descriptive material about families’ experiences of the whole care process rather than specifically on experience of volunteer care [5]. This demonstrates the utility of this quality assessment tool and also reflects the experiences of other researchers undertaking qualitative synthesis [47].

### Inclusion criteria

The inclusion of one study [3] also raises the question of whether our inclusion criteria should have excluded studies from developing countries, particularly given that the majority of available studies were from the developed world. Given that the synthesis method tends to privilege themes which recur within the available data, any study which stands out from the others is likely not to have much influence on the final themes reported. Although there may be a universal volunteer role common across cultures and healthcare systems, we consider that removing this study would not have benefited our research. An important reason why this study contributed so little was that the data collected by the authors was not analysed using the sophisticated methods employed in other studies rather than simply because it was from a developing country.

In addition, we excluded papers where the primary focus was on topics such as volunteer motivations, stress and burnout, and training, unless findings focused on care given by volunteers in patient/family-facing roles (Table 1). We appreciate that this may have led to our omitting relevant data but we needed to set realistic inclusion criteria because of the time-consuming nature of thematic synthesis. We consider that data saturation was reached since similar themes recurred in the included studies, so additional studies would not necessarily have added relevant new themes [48].

We also included studies with a range of respondents, volunteers, family members and volunteer co-ordinators. This allowed us to gain as wide an understanding as possible of how the volunteer role is conceived, rather than just one respondent group’s view, and whether some universal understanding of the role exists. It also acted as a triangulation method. The included studies were also from a variety of countries, just over half from North America and a third from Europe, as well as the one from Africa.

Another strength of our review was that we were able to explore the difference that care setting made on our findings, in particular the finding that our quasi-professional theme came mostly from studies based in inpatient care facilities and the surrogate family member theme from studies in home-based care.

The included studies employed a range of analysis methods which raises the issue of whether it is appropriate to combine data arising from different epistemological approaches. Little research has been undertaken on this issue [47] which largely remains theoretical. We could discern little difference between studies based on analysis method, except that those studies which did not state their analysis method contributed very little to the synthesis [5,33].

We analysed the questions asked by the researchers in our included studies when collecting their primary data, so far as the information in the published papers would allow. These show a wide range of topics covered, some directly relevant to our research question (for example, ‘Participants had the opportunity to share their….understanding of their role…’ [1], p.17, and less directly relevant (for example, ‘Opening question, "What made you become a volunteer here?". Then the volunteers were asked to describe personal experiences from their voluntary work.’ [29]). This demonstrates how a systematic review of qualitative findings can go beyond those findings, and even the original intentions, of individual studies to answer a separate research question.

### Thematic synthesis method

An important issue concerns the method of synthesis chosen, thematic synthesis, and how this suited our research question. Our question could be seen as being constructivist in nature, being about how the role of volunteer is understood. This could be construed as similar to how a role is constructed by relevant stakeholders, which is an idea based in an idealist epistemology (where reality is socially constructed [17]). However, our method draws on realist epistemology, where reality is considered knowable, albeit imperfectly [17,18]. However, methods for qualitative synthesis are underdeveloped and which method to apply in particular situations may become clearer [49].

In using thematic synthesis, we followed the method set out by Harden and Thomas [18]. However, this has previously been used mainly with relatively broad research questions that look at participant views on loosely defined topics (for example, children’s views on obesity [22] and barriers to, and facilitators of, healthy eating in children [50]). We, on the other hand, had a research question which was simultaneously less likely to be literally answerable from existing studies (from our searches of the wider literature on volunteers in palliative care settings we were not expecting to find studies which had focused specifically on how the volunteer role was understood) and was relatively tightly defined. Therefore, we kept our research question in mind throughout the synthesis process. This meant, for example, that when coding the studies for themes, we did not code literally every sentence of the findings or results sections as Thomas and Harden did [24]. Instead we coded only those portions of the text which were directly relevant to our research question, omitting sections which were clearly off-topic, such as some text on volunteer motivation [33], p.7].

In this way, the second stage of the thematic synthesis method as outlined by Thomas and Harden [24] was fulfilled, that is, developing descriptive themes. Thomas and Harden then describe using the descriptive themes to answer review questions by inferring answers from the themes, thus generating analytical themes. However, because of our constant mindfulness of our review question, we considered that we had effectively answered our question by the end of the second stage. We had largely focused on our review question when coding, for example, by omitting study findings which were clearly not relevant. Also, when comparing the codes generated in the initial coding and arranging them in a hierarchy we kept our research question in mind, asking ourselves, ‘What does this code tell us about the volunteer role?’. We believe that we have ‘gone beyond’ the original studies to produce a synthesis which does far more than merely describe the findings of the included studies.

## Conclusions

Given the aging population and current economic climate, it is likely that specialist palliative care will continue to rely on volunteer support. However, this should not imply that volunteers perform a substitutive role. Indeed, in the studies included in our review, volunteers appear to want to avoid this. As far as we are aware, this is the first systematic review of qualitative evidence to explore the volunteer role. We have shown how the volunteer role is understood, which will be useful for managers and others responsible for volunteer services or who work with volunteers to improve their everyday practice. We have also provided an example of the usefulness of thematic synthesis in synthesising qualitative studies.

## Competing interests

The authors declare that they have no competing interests.

## Authors’ contributions

BC, RR and RB conceived and designed the study; RB and BC acquired the data and undertook initial analysis; all authors were involved with additional analysis and interpretation of the data; RB drafted the article; all authors revised it critically for important intellectual content, and have read and approved the final version.

## Pre-publication history

The pre-publication history for this paper can be accessed here:

http://www.biomedcentral.com/1472-684X/13/3/prepub

## Supplementary Material

Additional file 1 Strings used to search electronic databases. Click here for file

Additional file 2 Characteristics of included studies. Click here for file

Additional file 3 Quality assessment of the included studies using the CASP criteria. Click here for file

Additional file 4 **Quality assessment of the included studies using additional criteria **[27,28]**.** Click here for file

Additional file 5 Themes found in included studies. Click here for file

Additional file 6 Example codes developed to generate a sub-theme. Click here for file
