# Supplementary material for: Understanding the role of the volunteer in specialist palliative care: a systematic review and thematic synthesis of qualitative studies
Source: BMC Palliat Care. 2014 Feb 10;13:3. doi: 10.1186/1472-684X-13-3 (PMC3928898; doi:10.1186/1472-684X-13-3)
Supplement: Additional file 1 — Strings used to search electronic databases. [file 1472-684X-13-3-S1.docx]

# Additional file 1: Strings used to search electronic databases

**Overall search strategy**

‘Volunteer’ AND ‘palliative’

Both free-text terms and subject headings tailored to each database, as follows:

**Free text string for ‘palliative’ used for all electronic databases**

| 1 | palliat*.mp. [mp=title, abstract, subject headings, heading word, drug trade name, original title, device manufacturer, drug manufacturer, device trade name, keyword] |
| --- | --- |
| 2 | terminal care.mp. |
| 3 | terminally ill.mp. |
| 4 | end-of-life care.mp. |
| 5 | end-of-life.mp. |
| 6 | hospice care.mp. |
| 7 | respite care.mp. |
| 8 | bereavement.mp. |
| 9 | grief.mp. |
| 10 | or/1-9 |

**Free text string for ‘volunteers’ used for all electronic databases**

| 1 | volunteer*.mp. [mp=title, abstract, subject headings, heading word, drug trade name, original title, device manufacturer, drug manufacturer, device trade name, keyword] |
| --- | --- |
| 2 | unpaid staff.mp. |
| 3 | lay worker.mp. |
| 4 | 1 or 2 or 3 |

**Amed MeSH terms for palliative**

| 1 | palliative care/ or terminal care/ |
| --- | --- |
| 2 | hospices/ |
| 3 | hospice care/ |
| 4 | respite care/ |
| 5 | exp bereavement/ |
| 6 | 1 or 2 or 3 or 4 or 5 |

**Amed MeSH terms for volunteer**

| 1 | voluntary workers/ |
| --- | --- |

**Embase MeSH terms for palliative**

| 1 | exp palliative therapy/ |
| --- | --- |
| 2 | exp terminal care/ |
| 3 | exp terminally ill patient/ |
| 4 | exp hospice care/ |
| 5 | exp hospice/ |
| 6 | exp bereavement/ or bereavement counseling/ |
| 7 | exp grief/ or exp anticipatory grief/ or exp mourning/ or exp sorrow/ |
| 8 | exp respite care/ |
| 9 | 1 or 2 or 3 or 4 or 5 or 6 or 7 or 8 |

**Embase MeSh terms for Volunteer**

| 1 | exp voluntary worker/ or volunteer/ |
| --- | --- |

**Medline MeSH terms for Palliative Care**

| 1 | exp palliative care/ |
| --- | --- |
| 2 | exp terminally ill/ |
| 3 | exp terminal care/ |
| 4 | exp hospice care/ |
| 5 | Hospices/ |
| 6 | exp bereavement/ |
| 7 | exp respite care/ |
| 8 | 1 or 2 or 3 or 4 or 5 or 6 or 7 |

**Medline MeSH terms for Volunteer**

| 1 | volunteer*.mp. [mp=title, abstract, subject headings, heading word, drug trade name, original title, device manufacturer, drug manufacturer, device trade name, keyword] |
| --- | --- |
| 2 | Hospital Volunteers/ |
| 3 | 1 or 2 |

**PsycInfo MeSh terms for Volunteer and Palliative care**

| 1 | "assistance (social behavior)"/ |
| --- | --- |
| 2 | charitable behavior/ |
| 3 | community involvement/ |
| 4 | cooperation/ |
| 5 | prosocial behavior/ |
| 6 | exp volunteers/ |
| 7 | 1 or 2 or 3 or 4 or 5 or 6 |
| 8 | exp Palliative Care/ |
| 9 | exp Terminally Ill/ |
| 10 | exp hospice/ |
| 11 | exp respite care/ |
| 12 | exp bereavement/ |
| 13 | 8 or 9 or 10 or 11 or 12 |

**Cinahl – terms for palliative care and volunteer:**

((((((Volunteer+Workers)+OR+(Volunteer+Experiences)))+OR+(volunteer)+OR+((lay+worker+OR+unpaid+staff+OR+unpaid+worker+OR+lay+staff))))+AND+(((((((Terminal+Care)+OR+(Hospice+Care)+OR+(Palliative+Care)))+OR+(((Respite+Care)))+OR+(((Bereavement)))))+OR+(((palliat*)+OR+(end-of life)+OR+(hospice)+OR+((terminal+care))+OR+((terminally+ill))+OR+((hospice+care))+OR+(bereavement))))))

**Dissertations and theses**

The search was run in stages because of the limitations of the database interface:

1. **[all(volunteer*) AND all((palliat* OR terminal care))](http://search.proquest.com/results.displayspellingsuggestions:dospellingsearch/$5bqueryType$3dadvanced:pqdt$3b+sortType$3drelevance$3b+searchTerms$3d$5b$3cAND$7call:volunteer*$3e,+$3cAND$7call:$28palliate*+OR+terminal+care$29$3e$5d$3b+searchParameters$3d$7bNAVIGATORS$3dsourcetypenav,pubtitlenav,languagenav$28filter$3d200$2f0$2f*$29,decadenav$28filter$3d110$2f0$2f*,sort$3dname$2fascending$29,yearnav$28filter$3d1100$2f0$2f*,sort$3dname$2fascending$29,yearmonthnav$28filter$3d120$2f0$2f*,sort$3dname$2fascending$29,monthnav$28sort$3dname$2fascending$29,daynav$28sort$3dname$2fascending$29,+RS$3dOP,+chunkSize$3d20,+ftblock$3d55000+1+55001,+DUPLICATIONREMOVAL$3dtrue$7d$3b+metaData$3d$7bUsageSearchMode$3dAdvanced,+dbselections$3ddissertations$7chealth,+siteLimiters$3dManuscriptType,_$25Language,+FDB$3dNONE$7d$5d?site=pqdt&t:ac=1345BE111AF23C156C2/1)**
2. **[all(volunteer*) AND all((terminally ill or end-of-life))](http://search.proquest.com/results.displayspellingsuggestions:dospellingsearch/$5bqueryType$3dadvanced:pqdt$3b+sortType$3drelevance$3b+searchTerms$3d$5b$3cAND$7call:volunteer*$3e,+$3cAND$7call:$28palliate*+OR+terminal+care$29$3e$5d$3b+searchParameters$3d$7bNAVIGATORS$3dsourcetypenav,pubtitlenav,languagenav$28filter$3d200$2f0$2f*$29,decadenav$28filter$3d110$2f0$2f*,sort$3dname$2fascending$29,yearnav$28filter$3d1100$2f0$2f*,sort$3dname$2fascending$29,yearmonthnav$28filter$3d120$2f0$2f*,sort$3dname$2fascending$29,monthnav$28sort$3dname$2fascending$29,daynav$28sort$3dname$2fascending$29,+RS$3dOP,+chunkSize$3d20,+ftblock$3d55000+1+55001,+DUPLICATIONREMOVAL$3dtrue$7d$3b+metaData$3d$7bUsageSearchMode$3dAdvanced,+dbselections$3ddissertations$7chealth,+siteLimiters$3dManuscriptType,_$25Language,+FDB$3dNONE$7d$5d?site=pqdt&t:ac=1345BE111AF23C156C2/1)**
3. **[all(volunteer*) AND all((hospice or respite))](http://search.proquest.com/results.displayspellingsuggestions:dospellingsearch/$5bqueryType$3dadvanced:pqdt$3b+sortType$3drelevance$3b+searchTerms$3d$5b$3cAND$7call:volunteer*$3e,+$3cAND$7call:$28palliate*+OR+terminal+care$29$3e$5d$3b+searchParameters$3d$7bNAVIGATORS$3dsourcetypenav,pubtitlenav,languagenav$28filter$3d200$2f0$2f*$29,decadenav$28filter$3d110$2f0$2f*,sort$3dname$2fascending$29,yearnav$28filter$3d1100$2f0$2f*,sort$3dname$2fascending$29,yearmonthnav$28filter$3d120$2f0$2f*,sort$3dname$2fascending$29,monthnav$28sort$3dname$2fascending$29,daynav$28sort$3dname$2fascending$29,+RS$3dOP,+chunkSize$3d20,+ftblock$3d55000+1+55001,+DUPLICATIONREMOVAL$3dtrue$7d$3b+metaData$3d$7bUsageSearchMode$3dAdvanced,+dbselections$3ddissertations$7chealth,+siteLimiters$3dManuscriptType,_$25Language,+FDB$3dNONE$7d$5d?site=pqdt&t:ac=1345BE111AF23C156C2/1)**
4. **[all(volunteer*) AND all((bereavement or grief))](http://search.proquest.com/results.displayspellingsuggestions:dospellingsearch/$5bqueryType$3dadvanced:pqdt$3b+sortType$3drelevance$3b+searchTerms$3d$5b$3cAND$7call:volunteer*$3e,+$3cAND$7call:$28palliate*+OR+terminal+care$29$3e$5d$3b+searchParameters$3d$7bNAVIGATORS$3dsourcetypenav,pubtitlenav,languagenav$28filter$3d200$2f0$2f*$29,decadenav$28filter$3d110$2f0$2f*,sort$3dname$2fascending$29,yearnav$28filter$3d1100$2f0$2f*,sort$3dname$2fascending$29,yearmonthnav$28filter$3d120$2f0$2f*,sort$3dname$2fascending$29,monthnav$28sort$3dname$2fascending$29,daynav$28sort$3dname$2fascending$29,+RS$3dOP,+chunkSize$3d20,+ftblock$3d55000+1+55001,+DUPLICATIONREMOVAL$3dtrue$7d$3b+metaData$3d$7bUsageSearchMode$3dAdvanced,+dbselections$3ddissertations$7chealth,+siteLimiters$3dManuscriptType,_$25Language,+FDB$3dNONE$7d$5d?site=pqdt&t:ac=1345BE111AF23C156C2/1)**

**Web of science**

The search was run in stages because of the limitations of the database interface:

1. **[(volunteer*) AND ((palliate* OR terminal care or](http://search.proquest.com/results.displayspellingsuggestions:dospellingsearch/$5bqueryType$3dadvanced:pqdt$3b+sortType$3drelevance$3b+searchTerms$3d$5b$3cAND$7call:volunteer*$3e,+$3cAND$7call:$28palliate*+OR+terminal+care$29$3e$5d$3b+searchParameters$3d$7bNAVIGATORS$3dsourcetypenav,pubtitlenav,languagenav$28filter$3d200$2f0$2f*$29,decadenav$28filter$3d110$2f0$2f*,sort$3dname$2fascending$29,yearnav$28filter$3d1100$2f0$2f*,sort$3dname$2fascending$29,yearmonthnav$28filter$3d120$2f0$2f*,sort$3dname$2fascending$29,monthnav$28sort$3dname$2fascending$29,daynav$28sort$3dname$2fascending$29,+RS$3dOP,+chunkSize$3d20,+ftblock$3d55000+1+55001,+DUPLICATIONREMOVAL$3dtrue$7d$3b+metaData$3d$7bUsageSearchMode$3dAdvanced,+dbselections$3ddissertations$7chealth,+siteLimiters$3dManuscriptType,_$25Language,+FDB$3dNONE$7d$5d?site=pqdt&t:ac=1345BE111AF23C156C2/1)****[terminally ill or end-of-life or](http://search.proquest.com/results.displayspellingsuggestions:dospellingsearch/$5bqueryType$3dadvanced:pqdt$3b+sortType$3drelevance$3b+searchTerms$3d$5b$3cAND$7call:volunteer*$3e,+$3cAND$7call:$28palliate*+OR+terminal+care$29$3e$5d$3b+searchParameters$3d$7bNAVIGATORS$3dsourcetypenav,pubtitlenav,languagenav$28filter$3d200$2f0$2f*$29,decadenav$28filter$3d110$2f0$2f*,sort$3dname$2fascending$29,yearnav$28filter$3d1100$2f0$2f*,sort$3dname$2fascending$29,yearmonthnav$28filter$3d120$2f0$2f*,sort$3dname$2fascending$29,monthnav$28sort$3dname$2fascending$29,daynav$28sort$3dname$2fascending$29,+RS$3dOP,+chunkSize$3d20,+ftblock$3d55000+1+55001,+DUPLICATIONREMOVAL$3dtrue$7d$3b+metaData$3d$7bUsageSearchMode$3dAdvanced,+dbselections$3ddissertations$7chealth,+siteLimiters$3dManuscriptType,_$25Language,+FDB$3dNONE$7d$5d?site=pqdt&t:ac=1345BE111AF23C156C2/1)****[hospice or respite](http://search.proquest.com/results.displayspellingsuggestions:dospellingsearch/$5bqueryType$3dadvanced:pqdt$3b+sortType$3drelevance$3b+searchTerms$3d$5b$3cAND$7call:volunteer*$3e,+$3cAND$7call:$28palliate*+OR+terminal+care$29$3e$5d$3b+searchParameters$3d$7bNAVIGATORS$3dsourcetypenav,pubtitlenav,languagenav$28filter$3d200$2f0$2f*$29,decadenav$28filter$3d110$2f0$2f*,sort$3dname$2fascending$29,yearnav$28filter$3d1100$2f0$2f*,sort$3dname$2fascending$29,yearmonthnav$28filter$3d120$2f0$2f*,sort$3dname$2fascending$29,monthnav$28sort$3dname$2fascending$29,daynav$28sort$3dname$2fascending$29,+RS$3dOP,+chunkSize$3d20,+ftblock$3d55000+1+55001,+DUPLICATIONREMOVAL$3dtrue$7d$3b+metaData$3d$7bUsageSearchMode$3dAdvanced,+dbselections$3ddissertations$7chealth,+siteLimiters$3dManuscriptType,_$25Language,+FDB$3dNONE$7d$5d?site=pqdt&t:ac=1345BE111AF23C156C2/1)** [or](http://search.proquest.com/results.displayspellingsuggestions:dospellingsearch/$5bqueryType$3dadvanced:pqdt$3b+sortType$3drelevance$3b+searchTerms$3d$5b$3cAND$7call:volunteer*$3e,+$3cAND$7call:$28palliate*+OR+terminal+care$29$3e$5d$3b+searchParameters$3d$7bNAVIGATORS$3dsourcetypenav,pubtitlenav,languagenav$28filter$3d200$2f0$2f*$29,decadenav$28filter$3d110$2f0$2f*,sort$3dname$2fascending$29,yearnav$28filter$3d1100$2f0$2f*,sort$3dname$2fascending$29,yearmonthnav$28filter$3d120$2f0$2f*,sort$3dname$2fascending$29,monthnav$28sort$3dname$2fascending$29,daynav$28sort$3dname$2fascending$29,+RS$3dOP,+chunkSize$3d20,+ftblock$3d55000+1+55001,+DUPLICATIONREMOVAL$3dtrue$7d$3b+metaData$3d$7bUsageSearchMode$3dAdvanced,+dbselections$3ddissertations$7chealth,+siteLimiters$3dManuscriptType,_$25Language,+FDB$3dNONE$7d$5d?site=pqdt&t:ac=1345BE111AF23C156C2/1) **[bereavement or grief))))](http://search.proquest.com/results.displayspellingsuggestions:dospellingsearch/$5bqueryType$3dadvanced:pqdt$3b+sortType$3drelevance$3b+searchTerms$3d$5b$3cAND$7call:volunteer*$3e,+$3cAND$7call:$28palliate*+OR+terminal+care$29$3e$5d$3b+searchParameters$3d$7bNAVIGATORS$3dsourcetypenav,pubtitlenav,languagenav$28filter$3d200$2f0$2f*$29,decadenav$28filter$3d110$2f0$2f*,sort$3dname$2fascending$29,yearnav$28filter$3d1100$2f0$2f*,sort$3dname$2fascending$29,yearmonthnav$28filter$3d120$2f0$2f*,sort$3dname$2fascending$29,monthnav$28sort$3dname$2fascending$29,daynav$28sort$3dname$2fascending$29,+RS$3dOP,+chunkSize$3d20,+ftblock$3d55000+1+55001,+DUPLICATIONREMOVAL$3dtrue$7d$3b+metaData$3d$7bUsageSearchMode$3dAdvanced,+dbselections$3ddissertations$7chealth,+siteLimiters$3dManuscriptType,_$25Language,+FDB$3dNONE$7d$5d?site=pqdt&t:ac=1345BE111AF23C156C2/1)**
2. **[all(volunteer*) AND all(()](http://search.proquest.com/results.displayspellingsuggestions:dospellingsearch/$5bqueryType$3dadvanced:pqdt$3b+sortType$3drelevance$3b+searchTerms$3d$5b$3cAND$7call:volunteer*$3e,+$3cAND$7call:$28palliate*+OR+terminal+care$29$3e$5d$3b+searchParameters$3d$7bNAVIGATORS$3dsourcetypenav,pubtitlenav,languagenav$28filter$3d200$2f0$2f*$29,decadenav$28filter$3d110$2f0$2f*,sort$3dname$2fascending$29,yearnav$28filter$3d1100$2f0$2f*,sort$3dname$2fascending$29,yearmonthnav$28filter$3d120$2f0$2f*,sort$3dname$2fascending$29,monthnav$28sort$3dname$2fascending$29,daynav$28sort$3dname$2fascending$29,+RS$3dOP,+chunkSize$3d20,+ftblock$3d55000+1+55001,+DUPLICATIONREMOVAL$3dtrue$7d$3b+metaData$3d$7bUsageSearchMode$3dAdvanced,+dbselections$3ddissertations$7chealth,+siteLimiters$3dManuscriptType,_$25Language,+FDB$3dNONE$7d$5d?site=pqdt&t:ac=1345BE111AF23C156C2/1)**
3. **[all(volunteer*) AND all(()](http://search.proquest.com/results.displayspellingsuggestions:dospellingsearch/$5bqueryType$3dadvanced:pqdt$3b+sortType$3drelevance$3b+searchTerms$3d$5b$3cAND$7call:volunteer*$3e,+$3cAND$7call:$28palliate*+OR+terminal+care$29$3e$5d$3b+searchParameters$3d$7bNAVIGATORS$3dsourcetypenav,pubtitlenav,languagenav$28filter$3d200$2f0$2f*$29,decadenav$28filter$3d110$2f0$2f*,sort$3dname$2fascending$29,yearnav$28filter$3d1100$2f0$2f*,sort$3dname$2fascending$29,yearmonthnav$28filter$3d120$2f0$2f*,sort$3dname$2fascending$29,monthnav$28sort$3dname$2fascending$29,daynav$28sort$3dname$2fascending$29,+RS$3dOP,+chunkSize$3d20,+ftblock$3d55000+1+55001,+DUPLICATIONREMOVAL$3dtrue$7d$3b+metaData$3d$7bUsageSearchMode$3dAdvanced,+dbselections$3ddissertations$7chealth,+siteLimiters$3dManuscriptType,_$25Language,+FDB$3dNONE$7d$5d?site=pqdt&t:ac=1345BE111AF23C156C2/1)**
4. **[all(volunteer*) AND all((or))](http://search.proquest.com/results.displayspellingsuggestions:dospellingsearch/$5bqueryType$3dadvanced:pqdt$3b+sortType$3drelevance$3b+searchTerms$3d$5b$3cAND$7call:volunteer*$3e,+$3cAND$7call:$28palliate*+OR+terminal+care$29$3e$5d$3b+searchParameters$3d$7bNAVIGATORS$3dsourcetypenav,pubtitlenav,languagenav$28filter$3d200$2f0$2f*$29,decadenav$28filter$3d110$2f0$2f*,sort$3dname$2fascending$29,yearnav$28filter$3d1100$2f0$2f*,sort$3dname$2fascending$29,yearmonthnav$28filter$3d120$2f0$2f*,sort$3dname$2fascending$29,monthnav$28sort$3dname$2fascending$29,daynav$28sort$3dname$2fascending$29,+RS$3dOP,+chunkSize$3d20,+ftblock$3d55000+1+55001,+DUPLICATIONREMOVAL$3dtrue$7d$3b+metaData$3d$7bUsageSearchMode$3dAdvanced,+dbselections$3ddissertations$7chealth,+siteLimiters$3dManuscriptType,_$25Language,+FDB$3dNONE$7d$5d?site=pqdt&t:ac=1345BE111AF23C156C2/1)**

**International Bibliography of the Social Sciences (IBSS), and Dissertations and Theses**

volunteer* AND (palliat* OR ("terminal care") OR ("terminally ill") OR ("end-of-life") OR (hospice) OR (respite) OR (bereavement) OR (grief))
