# Supplementary material for: Understanding the role of the volunteer in specialist palliative care: a systematic review and thematic synthesis of qualitative studies
Source: BMC Palliat Care. 2014 Feb 10;13:3. doi: 10.1186/1472-684X-13-3 (PMC3928898; doi:10.1186/1472-684X-13-3)
Supplement: Additional file 2 — Characteristics of included studies. [file 1472-684X-13-3-S2.docx]

Additional file 2 Characteristics of included studies

| **Study (year of publication)** | **Country** | **Setting** | **Study aims** | **Sample** | **Methods for data collection method/data analysis** |
| --- | --- | --- | --- | --- | --- |
| Andersson [29] | Sweden* | 3 Hospices | To understand what it means to be a hospice volunteer in a country without a tradition of hospice or palliative care volunteer services.  Opening question: what made you become a volunteer here? Then the volunteers were asked to describe personal experiences from their voluntary work. | Convenience sample of 10 volunteers (9 women, 1 man), aged 30-70, 4 retired, 6 months to 10 years’ experience as volunteer | Individual ‘narrative’ interviews/ phenomenological hermeneutic approach |
| Berry [30] | US | Hospice program, mostly home care. | 1. To investigate ethical issues that arise for hospice volunteers in working with patients, families, and caregivers at the end of life. 2. To identify general types of ethical situations or dilemmas experienced by hospice volunteers that fall outside of guidelines suggested by their volunteer training. 3. To learn more about the resources hospice volunteers use to manage ethical issues and challenges.  Opening statement about ethical issues. Questions:  1 Describe a situation in which you were not sure about the right of wrong thing to do from a moral or ethical point of view.  2 How did you feel about it?  3 What did you do?  4 Did you talk to anybody or consult any other sources of information? If so, please explain.  5 How was the issue resolved and if it was not, why not?  6 Say you are asked to help train the next group of volunteers. Dream big here. Tell us how you might better prepare volunteers to deal with issues similar to the ones you have described. | Convenience sample of 39 hospice volunteers (probably self-selected), mean age 64 years, 76% women, median 4 years’ hospice experience. 33 volunteered with large hospice program (home care) and 6 with smaller programs. | Semi-structured individual interviews/ thematic analysis |
| Field-Richards [31] | UK | Day care hospice | To understand volunteers’ experiences of working in a day care hospice with a particular emphasis on their perception of the boundaries that exist between paid and unpaid hospice workers.  No details of the questions asked other than the last question: Is there anything that we haven’t discussed which you feel is important in terms of your motivations and experiences as a volunteer? | Volunteers with patient-facing roles; self-selecting following distribution of invitation letters from volunteer coordinator; 12 participants, 10 female, 18-73 years, with 6 weeks to 6 years’ experience (mean 16 months) | Semi-structured individual interviews/ inductive and thematic analysis |
| Finn-Paradis [32] | US | Hospice program including home and inpatient care | To identify primary stressors experienced by volunteers providing direct patient care. However, data are from a study focusing on volunteer personality characteristics – they did telephone interviews with a subgroup of participants from wider study.  Four open-ended questions used to elicit concerns related to stress and burnout among volunteers. | 17 volunteers from hospice programs, aged 18-65 (mean 38) 10 women, plus unknown number spoken to at a conference | Telephone semi-structured interviews/ catalogued responses into 4 areas |
| Guirguis-Younger [1] | Canada | Hospice and acute-care hospital | To enrich our knowledge and understanding of the rewards, challenges, and unique commitments that define the experience of a palliative care volunteer  No details of the questions but ‘Participants had the opportunity to share their feelings, perceptions, and understanding of their role as a palliative care volunteer and its impact on their lives.’ | Volunteers from 3 palliative care settings – acute care hospital-based setting (7 participants, 5 women); freestanding community-funded hospice (6 women); shelter-based hospice facility for homeless (4 women) ; demographic data not collected but most participants in their 50s and 60s, and retired. | Focus groups/ grounded theory |
| Harris [33] | US | Home care | To determine differences and similarities in 1) the service provided by volunteers and that provided by paid workers; 2) characteristics brought to the respite situation by each group that had the potential to enhance the home care giving situation; and 3) quality of the relationship of the worker with the family  ‘…subjects were probed for their perceptions of worker motivation, role, and competence’ no further details. | 5 paid workers, 5 volunteers, (all 10 selected from larger study based on theoretical sampling technique (no details), plus families served by participants (number unclear). No demographic details of participants although mean age of those in larger study 51 years for volunteers and 38 for paid workers. Most participants were female. | Semi-structured individual interviews/no details |
| Jack [3] | Uganda* | Home care | To evaluate the impact of a palliative care community volunteer programme.  Separate questions for each sample – too many to list here (18 in all) but all pragmatic questions about volunteers. | Convenience sample of 21 patients (age 29-65 years, mean unclear because some patients unsure of their age) 15 women, 32 volunteers (age 28-52 years, mean 39) 17 women, 11 hospice clinical staff (no demographic details) | Semi-structured individual interviews and focus groups/ thematic analysis |
| Luijkx [5] | Holland | Home care | To understand the experiences of families of terminally ill patients with volunteer support.  What went right? What went wrong and what could be improved in the last phase of life?  What roles do professional caregivers and volunteers play in palliative care?  What are the implications of all of this for the quality of life of patients and their families? | Relatives of deceased former patients – 6 were interviewed (aged 34-82 years (mean not given)); 5 were women, and 22 took part in focus groups (aged 23-78 years; mean 56) 15 women | Semi-structured individual interviews and focus groups/no details |
| McKee [34] | Canada | Home care | To understand the role of hospice volunteers are in rural communities.  Questions:   - Please describe what volunteers do in your community? - What would be the ideal role of hospice volunteers in your community? - What are the motivations and expectations of your volunteers, and how do you address those in your community? - What differences do you perceive between hospice volunteering in rural as opposed to urban communities? | 13 Volunteer coordinators from all local independent hospice volunteer visiting programs (majority 55-64 years old; others 65+ - no further details); all women | Telephone interviews/grounded theory |
| Sevigny [35] | Canada | Home care | To understand and describe volunteer practices within palliative care system.  ‘Questions were developed to tackle all important themes linked to the study’s aim: volunteers’ motivations, objectives, tasks, and experiences. | 58 volunteers and 6 coordinators – 73% women, ages not given | Semi-structured individual interviews and focus groups/thematic analysis |
| Watts [36] | UK | Hospice | To understand the experience of becoming and being a hospice volunteer  Used topic guide for both focus group and interviews, but no further details. | 10 volunteers at 1 hospice; aged 41-71 years (no mean given); all women | Focus group and 2 semi-structured individual interviews/ thematic analysis |
| Weeks [37] | Canada | Home care | in this research we endeavoured to gain a clearer understanding of what impact hospice palliative care volunteers have on family caregivers.  ‘Questions on accessing services, services received, satisfaction, and experiences related to the volunteer’… | 10 bereaved women, aged 60-87 years, mean 74) | Semi-structured individual interviews/methodological hermeneutics |

* Data collected in local language although published report is in English
