# Supplementary material for: Understanding the role of the volunteer in specialist palliative care: a systematic review and thematic synthesis of qualitative studies
Source: BMC Palliat Care. 2014 Feb 10;13:3. doi: 10.1186/1472-684X-13-3 (PMC3928898; doi:10.1186/1472-684X-13-3)
Supplement: Additional file 3 — Quality assessment of the included studies using the CASP criteria. [file 1472-684X-13-3-S3.docx]

Additional Table 3 Quality assessment based on CASP criteria

| Study | **Clear statement of research aims** | **Qualitative methodology appropriate** | **Research design appropriate to address the aims of the research** | **Recruitment strategy appropriate to the aims of the research** | **Data collected in a way that addressed the research issue** | **Relationship between researcher and participants been adequately considered** | **Ethical issues been taken into consideration** | **Data analysis sufficiently rigorous** | **Clear statement of findings** |
| --- | --- | --- | --- | --- | --- | --- | --- | --- | --- |
| Andersson [29] | *✓* | *✓* | *✓* | *✓* | *✓* | *✓* | *✓* | X | *✓* |
| Berry [30] | *✓* | *✓* | *✓* | *✓* | *✓* | X | *✓* | *✓* | *✓* |
| Field-Richards [31] | *✓* | *✓* | *✓* | *✓* | *✓* | X | *✓* | *✓* | *✓* |
| Finn-Paradis [32] | *✓* | *✓* | *✓* | *✓* | *✓* | X | X | X | *✓* |
| Guirguis-Younger [1] | *✓* | *✓* | *✓* | *✓* | *✓* | *✓* | X | *✓* | *✓* |
| Harris [33] | *✓* | X | X | *✓* | X | X | X | X | *✓* |
| Jack [3] | *✓* | *✓* | *✓* | *✓* | *✓* | *✓* | X | *✓* | *✓* |
| Luijkx [5] | *✓* | *✓* | *✓* | *✓* | *✓* | X | X | X | *✓* |
| McKee [34] | *✓* | *✓* | *✓* | *✓* | *✓* | *✓* | *✓* | *✓* | *✓* |
| Sevigny [35] | *✓* | *✓* | *✓* | *✓* | *✓* | *✓* | *✓* | *✓* | *✓* |
| Watts [36] | *✓* | *✓* | *✓* | *✓* | *✓* | *✓* | *✓* | X | *✓* |
| Weeks [37] | *✓* | X | *✓* | *✓* | *✓* | *✓* | *✓* | *✓* | *✓* |
