# Supplementary material for: Understanding the role of the volunteer in specialist palliative care: a systematic review and thematic synthesis of qualitative studies
Source: BMC Palliat Care. 2014 Feb 10;13:3. doi: 10.1186/1472-684X-13-3 (PMC3928898; doi:10.1186/1472-684X-13-3)
Supplement: Additional file 4 — Quality assessment of the included studies using additional criteria [27,28]. [file 1472-684X-13-3-S4.docx]

Additional Table 4 Quality assessment of included studies using additional criteria

| **Title** | **Were steps taken to increase rigour in the sampling?** | **Were steps taken to increase rigour in the data collected?** | **Were steps taken to increase rigour in the analysis of the data?** | **Were findings of the study grounded in/supported by data?** | **Overall reliability/trustworthiness of the findings** | **Breadth and depth of findings?** | **To what extent does the study privilege the role of volunteers?** | **Overall usefulness** |
| --- | --- | --- | --- | --- | --- | --- | --- | --- |
| Andersson [29] | Several steps *3 hospices in Sweden chosen because of different organization and extent and orientation of volunteer services - no information on whether these were representative or not. Volunteers chosen based on convenient sampling [sic - should this be convenience sampling?].* | Several steps *Method suited research question.* | Several steps *Describe the method but unclear how researchers worked together, and extent to which they used opposing accounts to build the analysis.* | A few steps *Unclear how many volunteers contributed to quotes; sometimes quotes don't fit the point they are supposed to illustrate; 'themes' are very general and some of the material doesn't really fit them.* | *3/4* | Fair breadth/depth | To a large extent | 3/4 |
| Berry [30] | Several steps *Larger sample (n=39) although 30 from same hospice program. Demographics provided and appear representative of volunteers - eg mostly older, women, Caucasian. Unclear how recruited (other than though volunteer coordinators) but appear to be self-selected.* | A thorough attempt *Study focus was on ethical issues so focus of questions (given) was on that. Well described* | A thorough attempt *Well described; clear how both authors were involved and that they worked together.* | Several steps *Unclear how many different volunteers quotes are from.* | *3/4* | Fair breadth/depth *Findings clearly linked to original aim but there is sufficient breadth and depth.* | To a large extent | 2/4 |
| Field-Richards [31] | A few steps *Participants self-selecting, demographics similar to the average volunteer withe regard to gender (mostly women) but unclear on age and length of service, which varied from 6 weeks to 7 years (6 weeks seems too little but didn't have any exclusion criteria).* | Several steps *Semi-structured interviews using a prompt guide based on observations during informal hospice visits, adapted to each interviewee to ensure relevant topics discussed in depth.* | Several steps *Clear how reseachers worked together on the analysis and method followed.* | A thorough attempt | *3/4* | Good breadth/fair depth | To a large extent | 3/4 |
| Finn-Paradis [32] | Several steps *Volunters randomly selected (no details of procedure) from those involved in a study on volunteer personality characteristics based at one of 5 sites (mix of hospice programs). Don't seem typical volunteer profile - ony 59% women, mean age 38, only 2 didn't work full time, but all were white.* | A few steps | Not at all/not stated | A few steps | *2/4* | Fair breadth/depth | To a large extent | 3/4 |
| Guirguis-Younger [1] | Several steps *Focus groups from 3 different palliative care settings; participants seem typical of volunteers (older, white, female)* | A few steps | Several steps | Several steps | *3/4* | Fair breadth/depth | To a large extent | 3/4 |
| Harris [33] | A few steps *Convenience sample (unclear how this was made) fom whom 10 people were selected using theoretical sampling technique (unclear how this was done. Half of participants are paid workers, half volunteers, interviews also undertaken with families serviced by the participants.* | A few steps *In-depth interviews guided by a questionnaire - included demographic information and 'more penetrating questions about relationships and motivation', no further details.* | Not at all/not stated *No information on how qualitative data analysed other than 'qualitative data are presented in narrative and table form'* | A few steps *Very descriptive and not much real analysis.* | *2/4* | Fair breadth/depth | To some extent *Includes views of paid workers doing a similar role.* | 2/4 |
| Jack [3] | A thorough attempt | Several steps | Several steps | A thorough attempt | *3.5/4* | Good breadth/fair depth | To a large extent | 3/4 |
| Luijkx [5] | Several steps | A few steps *Nothing for focus groups, and a little for individual interviews* | Not at all/not stated | A few steps | *2/4* | Fair breadth/depth | To a large extent | 2/4 |
| McKee [34] | A thorough attempt *Interviewed 100% volunteer coordinators in one region of Ontario.* | Several steps *Clear interview schedule used, some indication of how interviews undertaken given.* | A thorough attempt | A thorough attempt | *3/4* | Good breadth/fair depth | To a large extent | 3/4 |
| Sevigny [35] | Several steps *Developed a theoretical sample, then sampled in 3 Canadian provinces - large number of respondents (n=64); representative of volunteers (eg mostly women); stopped recruiting when reached data saturation; unclear how volunteers chosen* | Several steps *Collection methods seem fine although no detail of the questions asked, they covered themes linked to the study aim.* | A few steps *Thematic analysis based on reference to paper in French. No mention of how themes tested or roles of different authors.* | A few steps *Findings seem to go beyond the data. Description of findings seem very overworked and overdeveloped with too much interpretation. Quotes are attributed to focus groups, interviews and workshop.* | *2.5/4* | Fair breadth/depth *There's quite a bit of depth and breadth but there is no acknowledement of complexity. All seems to fit and doesn't seem authentic.* | To a large extent | 3/4 |
| Watts [36] | A few steps *Unclear how the 8 participants were chosen from the 17 volunteers who expressed an interest in taking part. However, partaicipants represented a range of volunteer roles. Participants all from 1 hospice (this is a pilot study).* | Several steps *Undertook a focus group and 2 interviews using the focus group topic guide as an interview schedule.* | A few steps *Texts thematically analyzed drawing on some of the principles set out by Braun and Clarke (2006) - unclear what this involved. Analysis undertaken by a single researcher.* | A thorough attempt *Clear link between findings and data.* | *2.5/4* | Good breadth/depth | To a large extent | 4/4 |
| Weeks [37] | A thorough attempt  *Purposive sample supplied study team by paid coordinator of anonymised potential participants that included only the information necessary to draw a homogenous sample including age, length of HPC use, and length of time since their husband's death. List prioritized by the research team based on best fit to study criteria, was given back to the coordinator to ask the women's permission for a researcher to contact them. The first ten women to agree were interviewed.* | A thorough attempt | A thorough attempt | A thorough attempt | *4/4* | Good breadth/depth | To a large extent | 4/4 |
