# Supplementary material for: Understanding the role of the volunteer in specialist palliative care: a systematic review and thematic synthesis of qualitative studies
Source: BMC Palliat Care. 2014 Feb 10;13:3. doi: 10.1186/1472-684X-13-3 (PMC3928898; doi:10.1186/1472-684X-13-3)
Supplement: Additional file 5 — Themes found in included studies. [file 1472-684X-13-3-S5.docx]

Additional Table 5. Themes identified in each study

|  | **Andersson [29]*** | **Berry [30]*** | **Field-Richards [31]*** | **Finn Paradis[32]*** | **Guirguis-Younger [1]*** | **Harris [33]***** | **Jack [3]***** | **Luijkx [5]**** | **McKee 34]***** | **Sevigny [35]*** | **Watts [36]*** | **Weeks [37]**** |
| --- | --- | --- | --- | --- | --- | --- | --- | --- | --- | --- | --- | --- |
| Theme cluster: Distinctness of the volunteer role | | | | | | | | | | | | |
| Distinct role from staff | *✓* | *✓* |  |  |  |  |  |  | *✓* | *✓* |  | *✓* |
| Professional-like | *✓* | *✓* |  | *✓* | *✓* |  |  |  |  |  |  |  |
| Go-between |  |  | *✓* |  |  |  |  |  |  | *✓* |  |  |
| Advocate/mediator |  |  |  |  |  | *✓* |  |  |  | *✓* |  |  |
| Teamwork | *✓* |  | *✓* | *✓* | *✓* |  |  |  |  |  |  |  |
| Surrogate relationship roles |  |  | *✓* |  |  | *✓* |  |  | *✓* |  |  | *✓* |
| Theme cluster: Characteristics of the role | | | | | | | | | | | | |
| Social nature | *✓* | *✓* | *✓* | *✓* |  |  |  | *✓* | *✓* | *✓* | *✓* | *✓* |
| Providing support |  |  |  | *✓* |  |  |  | *✓* |  |  |  | *✓* |
| Just being there |  |  |  | *✓* | *✓* | *✓* |  |  | *✓* | *✓* |  | *✓* |
| Just listening |  | *✓* | *✓* |  |  |  |  |  | *✓* |  |  | *✓* |
| Keeping patients happy | *✓* |  |  |  |  |  |  |  | *✓* | *✓* |  | *✓* |
| Theme cluster: Volunteer experience of the role | | | | | | | | | | | | |
| Ambiguity |  |  |  | *✓* |  |  |  |  | *✓* |  |  |  |
| Flexibility |  | *✓* | *✓* |  | *✓* |  | *✓* |  | *✓* |  | *✓* | *✓* |
| Informality |  |  | *✓* |  | *✓* |  |  |  |  | *✓* | *✓* |  |
| Staff restrict information | *✓* |  | *✓* | *✓* |  |  |  |  |  |  |  |  |
| Staff control the role | *✓* |  | *✓* | *✓* |  |  |  |  |  |  |  |  |

* Respondents were volunteers; ** Respondents were family members; *** Respondents were mixed or had other roles

Shading indicates care setting: unshaded study based in hospice building; shaded home care only; vertical stripe mixed or unclear
