# Supplementary material for: Understanding the role of the volunteer in specialist palliative care: a systematic review and thematic synthesis of qualitative studies
Source: BMC Palliat Care. 2014 Feb 10;13:3. doi: 10.1186/1472-684X-13-3 (PMC3928898; doi:10.1186/1472-684X-13-3)
Supplement: Additional file 6 — Example codes developed to generate a sub-theme. [file 1472-684X-13-3-S6.doc]

**Additional file 6** **Example codes developed to generate a sub-theme**

| **Sub-theme** | **Codes used to develop this theme** |
| --- | --- |
| Distinct role from that of staff | Staff support  ‘Them and us’  Different role to staff  Volunteers enhance staff role  Boundaries evident between volunteers and staff |
| Specific volunteer roles: professional-like; go-between; advocate/mediator; teamwork; surrogate; relationship roles | Different volunteer roles (advocate/mediator)  Family surrogate  Para-professional role  Relationship roles |
| Social nature of the role | Relationships with families  - benefits for clients (social emotional benefits)  - nature of the relationship  - emotional or intimate  - importance of good relationships  - Examples of what they do  - social ‘tasks’  - emotional support  - developing relationships |
| Providing support | Emotional support |
| Just being there | Examples of what they do: being there  Nature of the role: being available  Downplaying the role |
| Just listening | Just listening  Downplaying the role |
| Keeping patients happy | Make patient happy  Examples of what they do: activities with patients |
| Ambiguity, flexibility and informality | Fill in the gaps  Uncertainty in role - unsure what to do  Informality attractive  Role is like a job  Role is not like a job  Changing nature of the role  Role isn’t fixed/role varies by individual  Ambiguous role or blurred boundaries between roles |
| Staff restrict information | Insufficient information about patients |
| Staff control the role | Reliance on staff for the role  Feel on the periphery |
